# *CTLA4* Haplotype Structures and −318 C>T (rs5742909) Genetic Variant Contribute to the Susceptibility of HPV Infection and Cervical Cancer

**DOI:** 10.3390/v17040453

**Published:** 2025-03-21

**Authors:** Maylla Cardoso de Oliveira, Janaina Nicolau de Oliveira, Eliza Pizarro Castilha, Giulia Mariane Fortunato, Pamella Rodrigues da Silva, Bianca Lisley Barboza Pacheco, José d’Oliveira Couto-Filho, Roberta Losi Guembarovski, Karen Brajão de Oliveira

**Affiliations:** 1 Laboratory of Molecular Genetics and Immunology, Department of Immunology, Parasitology and General Pathology, Center of Biological Sciences, State University of Londrina, Londrina 86057-970, PR, Brazil; maylla.oliveira@uel.br (M.C.d.O.); janaina.nicolau@uel.br (J.N.d.O.); eliza.pizarro.castilha@uel.br (E.P.C.); giulia.fortunato@uel.br (G.M.F.); pamella.rodrigues@uel.br (P.R.d.S.); bianca.pacheco@uel.br (B.L.B.P.); 2 Cancer Hospital of Londrina, Londrina 86015-520, PR, Brazil; clinicacouto@hotmail.com; 3 Department of General Biology, Biological Sciences Center, State University of Londrina, Londrina 86057-970, PR, Brazil; robertalosi@uel.br

**Keywords:** human papillomavirus, CD152, cytotoxic T-lymphocyte-associated antigen-4, cervical intraepithelial lesions, genetic polymorphism

## Abstract

High-risk Human Papillomavirus (HPV) infection is the main etiological factor for cervical carcinogenesis, although genetic cofactors also play a role. Single-nucleotide variants (SNVs) in the *CTLA4* gene can alter the gene expression and immune response against HPV, influencing cervical malignancy progression. This study analyzed the association of the alleles, genotypes, and haplotypes of the *CTLA4* SNVs rs5742909 (−318 C>T), rs231775 (+49 A>G), and rs3087243 (+6230 G>A) with HPV infection, the development of low-grade squamous intraepithelial lesions (LSILs), high-grade squamous intraepithelial lesions (HSILs), and cervical cancer in 445 women treated by the public health service of Paraná, Brazil. Peripheral blood and cervical secretion samples were collected for genomic DNA extraction, *CTLA4* SNV genotyping, and HPV detection via PCR. Statistical analyses used *p* < 0.05. The HPV-negative control group included 181 women, while the HPV-positive group included 264 women. The HPV-positive group was divided into no lesion (n = 84), LSILs (n = 19), HSILs (n = 56), and cervical cancer (n = 105). The T allele of −318 C>T and the TAG haplotype were associated with increased susceptibility to HPV infection, HSILs, and cervical cancer. These findings suggest that the T allele of −318 C>T and the TAG haplotype may serve as potential molecular biomarkers for HPV susceptibility and worse prognosis.

## 1. Introduction

The Human Papillomavirus (HPV) is considered the necessary etiological agent of cervical cancer, as it is responsible for around 99.7% of cases of cervical cancer [1]. Although infection with high-risk HPV (HR-HPV) is essential for carcinogenesis, it may not be sufficient, as the majority of infected individuals eliminate the virus without intervention [2]. However, there are also some cofactors, such as hormonal, immunologic, socioeconomic, environmental, and genetic cofactors [3].

CTLA-4 (cytotoxic T-lymphocyte associated protein 4) is an inhibitory receptor of the immune system that is involved in the downregulation of the T cell response and peripheral tolerance [4]. It is constitutively expressed on regulatory T cells (Tregs), but is also present on T CD4+ and T CD8+ cells [5]. Studies report that single-nucleotide variants (SNVs) in the gene encoding this receptor are associated with the susceptibility to autoimmune diseases and carcinogenesis [6,7].

*CTLA4* has more than one hundred genetic variants, but rs5742909 (−318 C>T), rs231775 (+49 A>G), and rs3087243 (+6230 G>A) have been the most intensively studied due to their potential effects on immune regulation [8]. Studies show that these genetic variants are associated with susceptibility to cervical cancer in different populations [9,10,11,12,13,14].

SNV rs5742909 (−318 C>T), which affects the *CTLA4* promoter sequence, is associated with increased promoter activity and, consequently, increased CTLA-4 expression on the cell surface, which decreases T cell activity [15]. In contrast, the SNV rs231775 (+49 A>G) causes the exchange of the amino acids threonine for alanine at position +49 of the CTLA-4 signaling peptide [16], resulting in the insufficient translocation of the growing CTLA-4 peptide from the ribosome to the endoplasmic reticulum lumen, and, consequently, the mis-targeting of CTLA-4 to the cell surface [17,18]. In addition, the SNV rs3087243 (+6230 G>A), which is present in the 3’ untranslated region of *CTLA4*, can affect the ratio of isoforms of this protein, decreasing the transcription of soluble CTLA-4 (sCTLA-4) and, consequently, increasing the transcription of full-length CTLA-4 (flCTLA-4), which is present in the cell membrane and which can decrease T cell activation [19].

The *CTLA4* genetic variants rs5742909 (−318 C>T), rs231775 (+49 A>G), and rs3087243 (+6230 G>A) have already been associated with the development of cervical cancer in different populations, but the results are contradictory. For example, when analyzing −318 C>T, Pawlak et al. and Xiong et al. demonstrated a significant association between the T allele and cervical cancer, while Rahimifar et al. found an association between the C allele and cervical cancer [12,13,14]. When analyzing +49 A>G, Pawlak et al., Rahimifar et al., and Xiong et al. found no association between this genetic variant and cervical cancer, while Gokhale et al. observed an association between the A allele and the development of cervical cancer [9,12,13,14]. In addition, Xiong et al. showed a significant association between the G allele of +6230 G>A and cervical cancer, while Gokhale et al. and Pawlak et al. found no association [9,12,14].

HPV is the most common sexually transmitted infection worldwide [20], while cervical cancer is the third most common cancer in Brazilian women [21]. Therefore, the study of possible associated genetic factors is essential for understanding the mechanisms of the carcinogenesis of cervical lesions. The *CTLA4* genetic variants rs5742909 (−318 C>T), rs231775 (+49 A>G), and rs3087243 (+6230 G>A) have not been previously associated with the development of cervical cancer in the Brazilian population, and the results of studies in other populations are contradictory, as shown. Therefore, the aim of this study was to investigate the association of the *CTLA4* genetic variants rs5742909 (−318 C>T), rs231775 (+49 A>G), and rs3087243 (+6230 G>A) with HPV infection, the development of cervical intraepithelial lesions, and cervical cancer in a cohort of women from the southern region of Brazil.

## 2. Materials and Methods

### 2.1. Ethical Approval and Sample Characterization

The present case-control study was approved by the Institutional Ethics Committee Involving Humans at the State University of Londrina, Londrina–Paraná (PR), Brazil (CAAE 38937520.2.0000.5231). The aim and steps of the study were explained to the patients and written informed consent was obtained. Each participant completed a structured questionnaire that included questions on sociodemographic data (age, smoking status, self-reported ethnicity, education level, marital status, and monthly income) and reproductive and sexual behavior (the use of oral contraceptives, use of condoms, age at menarche, age at first sexual intercourse, lifetime sexual partners, knowledge of HPV, knowledge of HPV transmission, previous cervical cancer screening, and a history of cervical cancer in the family).

The study analyzed 445 women who underwent outpatient cytology testing between 2013 and 2017 at the following healthcare facilities: an ambulatory colposcopy unit of Intermunicipal Consortium of Health of the Middle Paranapanema (CISMEPAR) in Londrina–PR, the Cancer Hospital of Londrina, the Outpatient Clinic of the University Hospital of Londrina, and two Basic Healthcare Units in Londrina–PR and Erasto Gaertner Hospital in Curitiba–PR. Following sample collection, cervical cells obtained using cytobrushes were stored in 2 mL of TE buffer (10 mM Tris-HCL, 1 mM EDTA, and a pH of 8.0) at −20 °C until DNA extraction. Additionally, peripheral blood samples from the participants were collected and stored at 7 °C in EDTA-containing tubes.

### 2.2. DNA Extraction and HPV Detection

The methods for the DNA extraction and HPV detection have been described in previous studies by our group [22,23].

### 2.3. Cervical Cytology

The cytological samples of the cervix were analyzed in the laboratories of the Public Health System of Londrina–PR and Curitiba–PR. The smears were classified as no lesions (cytologic samples were normal), low-grade squamous intraepithelial lesions (LSILs), high-grade squamous intraepithelial lesions (HSILs), or cervical cancer according to the Bethesda System (2014) [24].

### 2.4. CTLA4 Genetic Variants Genotyping

The identification of the genotypes of the *CTLA4* genetic variants (rs5742909 −318 C>T, rs231775 +49 A>G, and rs3087243 +6230 G>A) was performed by real-time PCR using validated assays (C__27834180_10, C___2415786_20, and C___3296043_10) from hydrolysis probes at 40× (TaqManTM System, Applied Biosystems, Walthan, MA, USA) diluted to 20× with high-purity water and stored at −20 °C. Reactions consisted of DNA (1 ng/μL), TaqMan™ Universal PCR Master Mix (TaqManTM System, Applied Biosystems, Walthan, MA, USA), and the specific probe for each genetic variant. Cycles were performed using the StepOne™ Real-Time PCR System Thermocycler (TaqManTM System, Applied Biosystems, Walthan, MA, USA).

### 2.5. Haplotype Analysis

The pairwise linkage disequilibrium (LD) was estimated using Haploview software version 4.2. The LD between the specified single-nucleotide variants (SNVs) was characterized using the D’ and r^2^ values. Recombination sites between the *CTLA4* alleles were inferred using PHASE software version 2.1.1, with haplotypes assigned based on maximum probability [25,26].

### 2.6. Statistical Analysis

Pearson’s chi-square test (χ^2^) was used to evaluate the differences in the frequency distributions of sociodemographic data and reproductive and sexual behavior characteristics, as well as *CTLA4* genetic variants (alleles, genotypes, and haplotypes), between the groups related to HPV infection and the lesion grades; however, when the expected frequency was less than 5, Fisher’s exact test was used instead of Pearson’s chi-square test. Regarding the *CLTA4* genetic variants genotypes, the dominant, codominant, and recessive genetic models were analyzed. The χ^2^ test was also used to evaluate the Hardy–Weinberg equilibrium. Data were expressed as absolute number (n) and percentage (%).

Binary logistic regression was performed to predict the independent associations between the variables and HPV infection group, while multinomial logistic regression was performed to predict the independent associations between the variables and cervical lesion study groups. Adjusted odds ratios (ORs) and 95% confidence intervals (CIs) were estimated. Statistical analyses were performed using the SPSS Statistics 25.0 software (SPSS, Inc., Chicago, IL, USA), considering the significance level of *p* < 0.05.

## 3. Results

The study population consisted of 445 women who were classified as HPV-infected (59.3%) and HPV-uninfected (HPV control group—40.7%). The HPV-infected women were divided into the following four groups based on the cervical cytology results: no lesion (31.8%), LSILs (7.2%), HSILs (21.2%), or cervical cancer (39.8%).

### 3.1. Association of Participant Sociodemographic, Reproductive, and Sexual Behavior Characteristics with HPV Infection and Cervical Lesion Status

It was observed that the age range (*p* < 0.001), smoking status (*p* = 0.042), educational level (*p* = 0.013), marital status (*p* = 0.006), monthly income (*p* = 0.023), oral contraceptive usage (*p* = 0.010), condom usage (*p* = 0.010), age at first sexual intercourse (*p* = 0.002), and number of sexual partners during the lifetime (*p* = 0.007) were associated with HPV infection. However, ethnicity (*p* = 0.773), age at menarche (*p* = 0.273), knowledge about HPV (*p* = 0.063), knowledge about HPV transmission (*p* = 0.124), previous cervical screening (*p* = 0.398), and familial cervical cancer (*p* = 0.958) were not associated with HPV infection (Supplementary Tables S1 and S2).

Binary logistic regression revealed a significantly higher proportion of infected women compared to uninfected women who were 24 years old or younger (Supplementary Tables S3 and S4), with these having 4.67 times higher odds of having HPV than those over 55 years old (OR = 4.67; CI 95% = 1.54–14.15). Women who had not completed high school were less likely to have HPV (OR = 0.36; CI 95% = 0.15–0.83) than those who had not completed elementary school. In addition, women who had one sexual partner in their lifetime were less likely to have HPV (OR = 0.43; CI 95% = 0.23–0.77) than women who had five or more partners in their lifetime.

Age (*p* < 0.001), education level (*p* < 0.001), marital status (*p* = 0.014), monthly income (*p* < 0.001), and knowledge of HPV transmission (*p* < 0.001) were associated with cervical lesion status. When this association was analyzed by adjusted multinomial logistic regression (Supplementary Table S5), the odds of having cervical cancer were lower among women who had not completed high school (OR = 0.050; CI 95% = 0.006–0.457) than among women who had not completed elementary school. Single women had a lower risk of developing LSILs (OR = 0.092; CI 95% = 0.013–0.656) than married women. It was also found that HPV-infected women who were 24 years old or younger had a 22.37-fold higher risk of developing LSILs than women over 55 years old (OR = 22.37; CI 95% = 1.55–322.123).

### 3.2. Distribution of Alleles, Genotypes, and Haplotypes of CTLA4 Genetic Variations and Susceptibility to HPV Infection, Cervical Lesions, and Cervical Cancer

The difference in the distribution of the rs5742909 (−318 C>T) genotype between the HPV infection groups was statistically significant for the codominant (*p* < 0.001), dominant (*p* = 0.004), and recessive (*p* = 0.020) genetic models (Table 1). Regarding the lesion grade groups, there was also a significant difference in the distribution in the dominant (*p* = 0.010) and codominant (*p* = 0.006) genetic models. Furthermore, a significant difference was observed in the distribution of rs5742909 (−318 C>T) alleles between the lesion grade groups (*p* = 0.012). The difference in the distribution of the rs3087243 (+6230 G>A) genotype between lesion grades was statistically significant in the codominant (*p* = 0.030) genetic model (Table 1).

Binary and multinomial logistic regressions were also performed to assess the impact of *CTLA4* SNVs on the susceptibility to HPV infection and the development of cervical lesions, respectively (Table 2). As for rs5742909 (−318 C>T), women with the CT genotype were more likely to be HPV-positive compared to those with the CC genotype (OR = 2.93; 95% CI = 1.71–5.02). In addition, women with TT+CT genotypes were more likely to have HPV infection than women with the CC genotype (OR = 2.38; 95% CI = 1.43–3.96). Women with the CT genotype were also more susceptible to developing HSILs (OR = 2.94; 95% CI = 1.27–6.80) and cervical cancer (OR = 4.20; 95% CI = 1.84–9.58) compared to women with the CC genotype. In the dominant genetic model, women with the TT+CT genotype were also more susceptible to developing HSILs (OR = 2.98; 95% CI = 1.26–6.76) and cervical cancer (OR = 4.31; 95% CI = 1.89–9.81) than women with the CC genotype.

Concerning rs3087243 (+6230 G>A), women with the AA+GA genotype were less likely to develop LSILs than women with the GG genotype (OR = 0.25; 95% CI = 0.06–0.95) (Table 2). In addition, women with the GA genotype were 3.48 times more susceptible to developing cervical cancer than women with the GG genotype (OR = 3.48; 95% CI = 1.33–9.05), while women with the AA genotype were less susceptible to developing cervical cancer than women with the GG+GA genotypes (OR = 0.28; 95% CI = 0.11–0.72). No statistical significance was found when analyzing the *CTLA4* genetic variant rs231775 (+49 A>G).

Combinations of the investigated genetic variants resulted in the following eight inferred haplotype structures: CAG, CAA, TAG, TAA, CGG, CGA, TGG, and TGA (composed of the genetic variants rs5742909 [−318 C>T], rs231775 [+49 A>G], and rs3087243 [+6230 G>A] of *CTLA4*). For the statistical analysis, only haplotypes with over a 5% frequency across the study population were tested; these included CAG, CAA, TAG, CGG, and TGG. The comparison of haplotype frequencies between the HPV infection and cervical lesion groups revealed no statistical differences (*p* > 0.05) (Table 3). However, the analysis of the association between *CTLA4* haplotypes and HPV infection identified a significant difference for the TAG haplotype (*p* = 0.007) (Table 4). Additionally, significant differences were observed in the haplotypic models of the CAA haplotype when associated with the development of cervical lesions (*p* = 0.004) (Table 5).

Logistic regression analysis was performed to determine whether the *CTLA4* haplotypes were independently associated with HPV infection and the development of cervical lesions (Table 6). Women who were heterozygous for the CAA haplotype had a lower risk of developing LSILs than women who did not carry this haplotype (OR = 0.25; 95% CI = 0.07–0.92). However, women heterozygous for the CAA haplotype had a 2.57 times higher risk of developing cervical cancer than women without this haplotype (OR = 2.57; 95% CI = 1.13–5.80). Regarding the TAG haplotype, heterozygous women were 2.41 times more likely to be HPV-positive than women without this haplotype (OR = 2.41; 95% CI = 1.36–4.25). In addition, women who were homozygous for TAG had an increased susceptibility to developing HSILs (OR = 2.56; 95% CI = 1.03–6.38) and cervical cancer (OR = 3.21; 95% CI = 1.34–7.69) compared to those who did not carry the TAG haplotype.

## 4. Discussion

To the best of our knowledge, this is the first study that associates the alleles, genotypes, and haplotypes of the *CTLA4* genetic variants rs5742909 (−318 C>T), rs231775 (+49 A>G), and rs3087243 (+6230 G>A) to HPV infection, the development of cervical lesions, and cervical cancer in Brazilian women.

Regarding traditional risk factors, we found that age, smoking status, education level, marital status, monthly income, oral contraceptive use, condom use, age at first sexual intercourse, and number of lifetime sexual partners were significantly associated with HPV infection. Age, education level, marital status, monthly income, and knowledge of HPV transmission were also variables associated with the development of cervical lesions or cervical cancer. Sociodemographic characteristics and sexual and reproductive behavior characteristics may also be cofactors associated with the development of HPV [27]. The observed data are consistent with the results of a previous study by our group [23].

In addition to HPV infection, genetic cofactors can also be associated with the development of cervical cancer. The CTLA-4 receptor plays a very important role in the immune system by negatively regulating T-lymphocytes [6,7]. Several genetic variants can affect *CTLA4* and lead to amino acid changes and alterations in messenger RNA (mRNA) splicing, which could affect T cell function and lead to a sustained attenuation of the immune response [28]. T cells play an essential role in the regression of HPV infections and cancer. However, *CTLA4* genetic variants can promote the downregulation of the T cell response and thus the progression of these diseases [29,30].

Our study demonstrated that the presence of the T allele related to rs5742909 (−318 C>T) may be a susceptibility factor for HPV infection, the development of HSILs, and cervical cancer. These data are consistent with previous studies linking the T allele of this genetic variant to the susceptibility to cervical cancer in different populations [11,12,14]. This genetic variation occurs in the *CTLA4* promoter region, which increases promoter activity and, consequently, CTLA-4 expression on the cell surface [15]. With the increased regulation of CTLA-4 on the surface of CD4+ or CD8+ T cells, this receptor binds in greater amounts to the B7 ligand present on antigen-presenting cells, such as dendritic cells during immunologic synapse, resulting in the downregulation of T cells [31,32]. A subpopulation of T cells, known as regulatory T cells, constitutively express CTLA-4 and secrete inhibitory cytokines, such as transforming growth factor beta (TGF-β) and interleukin-10, and also downregulate the activity of effector T cells [33,34]. T cells have important functions in fighting cancer and HPV infections [35]. However, when their function is impaired due to immunosuppression caused by the overactivation of CTLA-4, such as genetic variants, progression of the malignant process may occur.

When we analyzed rs3087243 (+6230 G>A), we found that the presence of the A allele may be a protective factor for LSILs; however, the A allele may also be a susceptibility factor for the development of cervical cancer. In addition to CTLA-4, there are other molecules that play an important role in regulating the T cell response, such as CD28 and inducible costimulator (ICOS), which are considered costimulatory molecules. The genes encoding CD28, CTLA-4, and ICOS are located next to each other in a 300 kb long segment in the q33 region of chromosome 2 [36]. Therefore, we hypothesized that genetic variants in the genes encoding CD28 and/or ICOS may have been inherited together due to their proximity on the chromosome and possibly affect the functional action of *CTLA4* rs3087243 (+6230 G>A), but studies are still needed for a better understanding.

The haplotype analyses made it possible to observe the association of the TAG haplotype with HPV infection and the association of the CAA, TAG, and TGG haplotypes with the status of the cervical lesion. Logistic regression, eliminating potential confounders, found that the presence of the TAG haplotype could be a susceptibility factor for HPV infection, the development of HSILs, and cervical cancer. This finding leads to hypotheses about the heritability of the TAG haplotype as a factor associated with cervical cancer. Although the A allele of rs3087243 (+6230 G>A) is present in the TAG haplotype and was also associated with cervical cancer in our study, the OR of association of the CT genotype of rs5742909 (−318 C>T) with cervical cancer is much higher. It is therefore suggested that the presence of the T allele of rs5742909 (−318 C>T) promotes the association of the TAG haplotype with the susceptibility to cervical cancer development.

Previous studies have investigated *CTLA4* haplotypes in different populations. When analyzing the haplotypes of the *CTLA4* genetic variants rs5741909 (−318 C>T), rs231775 (+49 A>G), and rs3087243 (+6230 G>A), Su et al. and Gokhale et al. found no statistical association with cervical cancer [9,37]. However, a study examining haplotypes composed of the *CTLA4* genetic variants −1722 (C>T), −1661 (A>G), −318 (C>T), and +49 (A>G) found that the presence of the TGCG haplotype may be associated with an increased risk of cervical cancer [13]. In our allele and genotype analyses, no statistical significance was found when analyzing the association between rs231775 (+49 A>G) and HPV infection, the development of cervical lesions, or cervical cancer. However, in the haplotype analyses, the G allele of rs231775 (+49 A>G) was present in the TAG haplotype combination associated with HPV infection, the development of HSILs, and cervical cancer. Therefore, our data are consistent with the study by Rahimifar et al., who studied the Iranian population [13]. However, further studies need to be conducted to confirm the hypothesis that the genetic variant rs231775 (+49 A>G) could be a susceptibility factor for the development of cervical cancer in the Brazilian population.

This study has some limitations, such as the number of samples, especially in the LSIL group, which may reduce the statistical power of our analysis. The lower number of samples in the LSIL group may have been caused by the fact that cervical lesions can develop directly into HSILs without LSILs occurring [38,39]. However, a plus point is that we collected sociodemographic data as well as data on the sexual and reproductive behavior of the participants, which allowed for an analysis of the possible confounding factors in the study of *CTLA4* genetic variants potentially associated with cervical cancer. It is noteworthy that studies involving women from other regions of Brazil are necessary to obtain more reliable results given the heterogeneity of the country.

In brief, we have shown that allele T of the *CTLA4* genetic variant rs5742909 (−318 C>T) and the TAG haplotype are associated with HPV infection, the development of cervical intraepithelial lesions, and cervical cancer in a cohort of the Brazilian population. Our study therefore suggests that this genetic variant and haplotype may be relevant molecular biomarkers for susceptibility to HPV infection and worst prognosis.

## Figures and Tables

**Table 1:** Association of the *CTLA4* genetic variants rs5742909 (−318 C>T), rs231775 (+49 A>G), and rs3087243 (+6230 G>A) with HPV infection and cervical lesion status.

Genetic Models		HPV					Lesion Grade (HPV-Infected Patients)								
		Uninfected (n = 181)		Infected (n = 264)		*p*-Value	NL (n = 84)		LSILs (n = 19)		HSILs (n = 56)		CC (n = 105)		*p*-Value
		N	%	N	%		N	%	N	%	N	%	N	%	
rs5742909 (−318 C>T)															
Codominant	CC	148	82.2	185	70.3	<0.001 *	70	83.3	15	78.9	36	64.3	64	61.5	0.010 *
	CT	26	14.4	77	29.3		14	16.7	4	21.1	20	35.7	39	37.5	
	TT	6	3.4	1	0.4		0	0.0	0	0.0	0	0.0	1	1.0	
Dominant	CC	148	82.2	185	70.3	**0.004**	70	83.3	15	78.9	36	64.3	64	61.5	**0.006**
	CT+TT	32	17.8	78	29.7		14	16.7	4	21.1	20	35.7	40	38.5	
Recessive	CC+CT	174	96.7	262	99.6	0.020 *	84	100	19	100	56	100	103	99.0	1.000 *
	TT	6	3.3	1	0.4		0	0.0	0	0.0	0	0.0	1	1.0	
Alleles	C	322	89.5	447	84.9	3.714	154	74.4	34	89.5	92	82.1	167	80.3	**0.012**
	T	38	10.5	79	15.1		14	25.6	4	10.5	20	17.9	41	19.7	
rs231775 (+49 A>G)															
Codominant	AA	66	36.5	100	38.0	0.369	33	39.3	4	21.1	21	37.5	42	40.4	0.715
	AG	86	47.5	133	50.6		44	52.4	12	63.2	28	50.0	49	47.1	
	GG	29	16.0	30	11.4		7	8.3	3	15.7	7	12.5	13	12.5	
Dominant	AA	66	36.5	100	38.0	0.739	33	39.3	4	21.1	21	37.5	42	40.4	0.452
	AG+GG	115	63.5	163	62.0		51	60.7	15	78.9	35	62.5	62	59.6	
Recessive	AA+AG	152	84.0	233	88.6	0.159	77	91.7	16	84.2	49	87.5	91	87.5	0.721
	GG	29	16.0	30	11.4		7	8.3	3	15.8	7	12.5	13	12.5	
Alleles	A	218	60.2	333	63.3	0.351	110	65.5	20	52.6	70	62.5	133	63.9	0.517
	G	144	39.8	193	36.7		58	34.5	18	47.4	42	37.5	75	36.1	
rs3087243 (+6230 G>A)															
Codominant	GG	51	31.8	58	26.4	0.344	19	28.8	8	50.0	11	27.5	20	20.5	**0.030**
	GA	79	49.4	125	56.8		30	45.5	6	37.5	23	57.5	66	67.3	
	AA	30	18.8	37	16.8		17	25.7	2	12.5	6	15.0	12	12.2	
Dominant	GG	51	31.9	58	26.4	0.241	19	28.8	8	50.0	11	27.5	20	20.4	0.085
	GA+AA	109	68.1	162	73.6		47	71.2	8	50.0	29	72.5	78	79.6	
Recessive	GG+GA	130	81.2	183	83.2	0.626	49	74.2	14	87.5	34	85.0	86	87.8	0.136
	AA	30	18.8	37	16.8		17	25.8	2	12.5	6	15.0	12	12.2	
Alleles	G	181	56.6	241	54.8	0.623	68	51.5	22	68.7	45	56.3	106	62.7	0.362
	A	139	43.4	199	45.2		64	48.5	10	31.3	35	43.7	90	37.3	

Data are presented as the absolute number and percentage. Analysis was carried out using the two-tailed chi-square (X^2^) test or * Fisher’s test, with *p* < 0.05 being adopted as the significance level (SPSS Inc., Chicago, IL, USA). HPV (Human Papillomavirus); NL (no lesion); LSILs (low-grade squamous intraepithelial lesions); HSILs (high-grade squamous intraepithelial lesions); CC (cervical cancer). Bold values represent statistical significance (*p* < 0.05). The distribution of the genotype frequency of rs5742909 (−318 C>T) was not in the Hardy–Weinberg equilibrium on HPV-uninfected group (*p* < 0.05). Some of the participants did not complete the genotyping for all of the genetic variants analyzed.

**Table 2:** Association of the *CTLA4* genetic variants rs5742909 (−318 C>T), rs231775 (+49 A>G), and rs3087243 (+6230 G>A) with HPV infection and cervical lesion status through adjusted logistic regression.

Genetic Models		HPV		Lesion Grade (HPV-Infected Patients)					
		Infected	Adj. *p*-Value	LSILs	Adj. *p*-Value	HSILs	Adj. *p*-Value	CC	Adj. *p*-Value
		OR (CI95%)		OR (CI95%)		OR (CI95%)		OR (CI95%)	
rs5742909 (−318 C>T)									
Codominant	CC	Reference		Reference		Reference		Reference	
	CT	2.936 (1.715–5.026)	**<0.001**	1.057 (0.277–4.035)	0.935	2.945 (1.274–6.808)	**0.012**	4.207 (1.846–9.588)	**0.001**
	TT	-	0.999	-	1	-	1	-	0.997
Dominant	CC	Reference		Reference		Reference		Reference	
	TT+CT	2.382 (1.432–3.963)	**0.001**	1.057 (0.277–4.035)	0.935	2.928 (1.267–6.766)	**0.012**	4.314 (1.896–9.817)	**<0.001**
Recessive	CC+CT	Reference		Reference		Reference		Reference	
	TT	-	0.999	-	1	-	1	-	0.997
rs231775 (+49 A>G)									
Codominant	AA	Reference		Reference		Reference		Reference	
	AG	0.940 (0.594–1.486)	0.790	2.352 (0.623–8.876)	0.207	1.209 (0.554–2.641)	0.634	1.106 (0.538–2.273)	0.785
	GG	0.712 (0.366–1.384)	0.316	4.135 (0.568–30.090)	0.161	2.179 (0.625–7.597)	0.221	1.298 (0.397–4.239)	0.666
Dominant	AA	Reference		Reference		Reference		Reference	
	GG+AG	0.883 (0.572–1.365)	0.576	2.573 (0.702–9.425)	0.154	1.346 (0.637–2.843)	0.437	1.127 (0.565–2.248)	0.734
Recessive	AA+AG	Reference		Reference		Reference		Reference	
	GG	0.737 (0.399–1.360)	0.329	2.327 (0.409–13.228)	0.341	1.954 (0.607–6.288)	0.261	1.223 (0.401–3.733)	0.723
rs3087243 (+6230 G>A)									
Codominant	GG	Reference		Reference		Reference		Reference	
	GA	1.190 (0.707–2.003)	0.512	0.299 (0.073–1.227)	0.094	1.098 (0.391–3.090)	0.859	3.481 (1.338–9.056)	**0.011**
	AA	1.023 (0.521–2.011)	0.947	0.181 (0.026–1.264)	0.085	0.523 (0.144–1.895)	0.323	0.641 (0.207–1.983)	0.440
Dominant	GG	Reference		Reference		Reference		Reference	
	AA+GA	1.144 (0.698–1.875)	0.594	0.250 (0.065–0.955)	**0.043**	0.891 (0.335–2.367)	0.817	2.111 (0.878–5.077)	0.095
Recessive	GG+GA	Reference		Reference		Reference		Reference	
	AA	0.918 (0.508–1.661)	0.777	0.413 (0.074–2.294)	0.312	0.485 (0.159–1.478)	0.203	0.283 (0.111–0.724)	**0.008**

Data were analyzed by logistic regression adjusted for “age range”, “educational level”, and “sexual partners during the lifetime” (HPV infection analysis) or “age range”, “education level”, and “marital status” (lesion grade analysis), with the “uninfected” group or “no lesion” group as the reference, respectively, and with *p* < 0.05 considered significant (bold) (SPSS Inc., Chicago, Illinois, USA). HPV (Human Papillomavirus); LSILs (low-grade squamous intraepithelial lesions); HSILs (high-grade squamous intraepithelial lesions); CC (cervical cancer); OR (odds ratio); CI (confidence interval); Adj. (adjusted). Some of the variable categories were excluded from the logistic regression, likely due to sample size limitations.

**Table 3:** Association of *CTLA4* haplotypes composed of the genetic variants rs5742909 (−318 C>T), rs231775 (+49 A>G), and rs3087243 (+6230 G>A) with HPV infection and cervical lesion status.

*CTLA4* Haplotypes	HPV					Lesion Grade (HPV-Infected Patients)								
	Uninfected (n = 362)		Infected (n = 528)		*p*-Value	No lesion (n = 168)		LSILs (n = 38)		HSILs (n = 112)		CC (n = 210)		*p*-Value
	N	%	N	%		N	%	N	%	N	%	N	%	
CAG	41	47.7	45	52.3	0.071	16	9.6	4	10.2	11	10.1	14	6.7	0.1
CAA	152	40.2	226	59.8		81	48.5	12	30.7	42	38.5	91	43.7	
TAG	25	28.7	62	71.3		12	7.2	4	10.2	17	15.6	29	13.9	
CGG	129	42.3	176	57.7		57	34.1	18	46.4	37	33.9	64	30.8	
TGG	14	51.8	13	48.2		1	0.6	0	2.5	2	1.9	10	4.9	

Data are presented as the absolute number and percentage. Analysis was carried out using the two-tailed chi-square (Χ^2^) test, with *p* < 0.05 being adopted as the significance level (SPSS Inc., Chicago, IL, USA). HPV (Human Papillomavirus); LSILs (low-grade squamous intraepithelial lesions); HSILs (high-grade squamous intraepithelial lesions); CC (cervical cancer). The “n” value corresponds to the total number of chromosomes analyzed and the “N” value corresponds to the number of chromosomes containing the haplotypes. SNV alleles in the haplotype structures follow the order rs231775 A>G, rs5742909 C>T, and rs3087243 G>A.

**Table 4:** Association of the haplotype models of the *CTLA4* genetic variants rs5742909 (−318 C>T), rs231775 (+49 A>G), and rs3087243 (+6230 G>A) with HPV infection.

Haplotype Models	HPV − (n = 181)		HPV + (n = 264)		*p*-Value
	N	%	N	%	
CAG/CAG	4	2.2	4	1.6	0.449
CAG/OTHERS	33	18.3	37	14.3	
OTHERS/OTHERS	143	79.5	217	84.1	
CAA/CAA	34	18.9	45	17.4	0.550
CAA/OTHERS	84	46.7	134	51.9	
OTHERS/OTHERS	62	34.4	79	30.7	
TAG/TAG	1	0.6	0	0.0	**0.007**
TAG/OTHERS	23	12.8	62	24.0	
OTHERS/OTHERS	156	86.6	196	76.0	
CGG/CGG	21	11.7	21	8.1	0.462
CGG/OTHERS	86	47.8	130	50.4	
OTHERS/OTHERS	73	40.5	107	41.5	
TGG/TGG	4	2.2	1	0.4	0.185
TGG/OTHERS	6	3.3	11	4.3	
OTHERS/OTHERS	170	94.5	246	95.3	

Data are presented as the absolute number and percentage. Analysis was carried out using the two-tailed chi-square (Χ^2^) test, with *p* < 0.05 being adopted as the significance level (SPSS Inc., Chicago, IL, USA). HPV (Human Papillomavirus). Bold values represent statistical significance (*p* < 0.05). SNV alleles in haplotype structures follow the order rs5742909 (−318 C>T), rs231775 (+49 A>G), and rs3087243 (+6230 G>A). Some of the participants did not complete genotyping for all of the genetic variants analyzed, so their haplotypes were incomplete and they were not counted.

**Table 5:** Association of haplotype models of *CTLA4* genetic variants rs5742909 (−318 C>T), rs231775 (+49 A>G), and rs3087243 (+6230 G>A) with cervical lesion status.

Haplotype Models	NL (n = 84)		LSILs (n = 19)		HSILs (n = 56)		CC (n = 105)		*p*-Value
	N	%	N	%	N	%	N	%	
CAG/CAG	2	2.4	0	0	1	1.9	1	1.0	0.859
CAG/OTHERS	12	14.5	4	21.1	9	17.0	12	11.7	
OTHERS/OTHERS	69	83.1	15	78.9	43	81.1	90	87.3	
CAA/CAA	21	25.3	3	15.8	10	18.9	11	10.7	**0.004**
CAA/OTHERS	38	45.8	6	31.6	22	41.5	68	66.0	
OTHERS/OTHERS	24	28.9	10	52.6	21	39.6	24	23.3	
TAG/TAG	0	0	0	0	0	0	0	0	0.069
TAG/OTHERS	12	14.5	4	21.1	17	32.1	29	28.2	
OTHERS/OTHERS	71	85.5	15	78.9	36	67.9	74	71.8	
CGG/CGG	7	8.4	3	15.8	3	5.7	8	7.8	0.471
CGG/OTHERS	43	51.8	12	63.2	28	52.8	47	45.6	
OTHERS/OTHERS	33	39.8	4	21.0	22	41.5	48	46.6	
TGG/TGG	0	0	0	0	0	0	1	1.0	0.280
CGG/OTHERS	1	1.2	0	0	2	3.8	8	7.8	
OTHERS/OTHERS	82	98.8	19	100.0	51	96.2	94	91.2	

Data presented as the absolute number and percentage. Analysis was carried out using the two-tailed chi-square (Χ^2^) test, with *p* < 0.05 being adopted as the significance level (SPSS Inc., Chicago, IL, USA). Bold values represent statistical significance (*p* < 0.05). SNV alleles in haplotype structures follow the order rs5742909 (−318 C>T), rs231775 (+49 A>G), and rs3087243 (+6230 G>A). NL (no lesion); CC (cervical cancer). Some of the participants did not complete genotyping for all of the analyzed genetic variants, resulting in incomplete haplotypes that were excluded from the analysis.

**Table 6:** Association of *CTLA4* haplotypes composed of the genetic variants rs5742909 (−318 C>T), rs231775 (+49 A>G), and rs3087243 (+6230 G>A) with HPV infection and cervical lesion status through adjusted logistic regression.

Haplotype Models	HPV		Lesion Grade (HPV-Infected Patients)					
	Infected	Adj. *p*-Value	LSILs	Adj. *p*-Value	HSILs	Adj. *p*-Value	CC	Adj. *p*-Value
	OR (CI95%)		OR (CI95%)		OR (CI95%)		OR (CI95%)	
CAG								
CAG/CAG	0.702 (0.146–3.377)	0.659	-	0.999	0.758 (0.061–9.368)	0.829	0.123 (0.009–1.696)	0.118
CAG/OTHERS	0.877 (0.499–1.540)	0.648	1.954 (0.515–7.410)	0.325	1.269 (0.460–3.506)	0.646	0.669 (0.247–1.811)	0.429
OTHERS/OTHERS	Reference		Reference		Reference		Reference	
CAA								
CAA/CAA	0.977 (0.532–1.796)	0.941	0.295 (0.066–1.327)	0.112	0.479 (0.178–1.289)	0.145	0.471 (0.165–1.344)	0.159
CAA/OTHERS	1.026 (0.638–1.650)	0.916	0.258 (0.072–0.924)	**0.037**	0.532 (0.226–1.255)	0.150	2.571 (1.139–5.803)	**0.023**
OTHERS/OTHERS	Reference		Reference		Reference		Reference	
TAG								
TAG/TAG	-	1	1.907 (0.502–7.242)	0.343	2.569 (1.033–6.388)	**0.042**	3.212 (1.340–7.695)	**0.009**
TAG/OTHERS	2.414 (1.368–4.258)	**0.002**	-	-	-	-	-	-
OTHERS/OTHERS	Reference		Reference		Reference		Reference	
CGG								
CGG/CGG	0.627 (0.295–1.332)	0.225	4.608 (0.742–28.610)	0.101	0.950 (0.209–4.316)	0.947	0.629 (0.169–2.340)	0.490
CGG/OTHERS	0.901 (0.575–1412)	0.650	2.215 (0.610–8.045)	0.227	1.109 (0.508–2.422)	0.794	0.900 (0.446–1.819)	0.770
OTHERS/OTHERS	Reference		Reference		Reference		Reference	
TGG								
TGG/TGG	-	0.999	-	-	-	1	-	0.998
TGG/OTHERS	1.882 (0.633–5.596)	0.255	-	0.996	4.240 (0.357–50.394)	0.253	9.210 (0.809–104.901)	0.074
OTHERS/OTHERS	Reference		Reference		Reference		Reference	

Data were analyzed by logistic regression adjusted for “age range”, “educational level”, and “sexual partners during the lifetime” (HPV infection analysis) or “age range”, “education level”, and “marital status” (lesion grade analysis), with the “uninfected” group or “no lesion” groups as the reference, respectively, and with *p* < 0.05 considered significant (bold) (SPSS Inc., Chicago, IL, USA). HPV (Human Papillomavirus); LSILs (low-grade squamous intraepithelial lesions); HSILs (high-grade squamous intraepithelial lesions); CC (cervical cancer); OR (odds ratio); CI (confidence interval); Adj. (adjusted). Some categories of the variables did not complete the logistic regression, probably due to the sample size.

## Data Availability

The original contributions presented in this study are included in the article/Supplementary Materials. Further inquiries can be directed to the corresponding author(s).

## Supplementary Materials

The following supporting information can be downloaded at: https://www.mdpi.com/article/10.3390/v17040453/s1, Table S1: Association of participant sociodemographic characteristics with HPV infection and cervical lesion status; Table S2: Association of participant reproductive and sexual behavior characteristics with HPV infection and cervical lesion status; Table S3: Association of participant sociodemographic characteristics with HPV infection through adjusted logistic regression; Table S4: Association of participant reproductive and sexual behavior characteristics with HPV infection through adjusted logistic regression; Table S5: Association of participant sociodemographic, reproductive, and sexual behavior characteristics with cervical lesion groups through adjusted logistic regression.
