# Supplementary material for: CTLA4 Haplotype Structures and −318 C>T (rs5742909) Genetic Variant Contribute to the Susceptibility of HPV Infection and Cervical Cancer
Source: Viruses. 2025 Mar 21;17(4):453. doi: 10.3390/v17040453 (PMC12031065; doi:10.3390/v17040453)
Supplement: Supplementary file 1 [file viruses-17-00453-s001.zip › Supplementary Table S1.pdf]

Supplementary Table S1. Association of participant sociodemographic characteristics with HPV infection and cervical lesion status.

Variables		HPV				*p*-value	Lesion grade (HPV infected patients)								*p*-value
		Uninfected (n=181)		Infected (n=264)			NL (n=84)		LSIL (n=19)		HSIL (n=56)		CC (n=105)		
		N	%	N	%		N	%	N	%	N	%	N	%	
Age range (years)	≤ 24	9	5.0	33	12.5	**<0.001**	14	16.7	6	31.6	13	23.2	0	0.0	**<0.001***
	25 – 34	42	23.2	58	22.0		28	33.3	6	31.6	15	26.8	9	8.6	
	35 – 44	45	24.9	58	22.0		17	20.2	3	15.8	12	21.4	26	24.8	
	45 – 54	57	31.5	47	17.8		13	15.5	3	15.8	10	17.9	21	20.0	
	≥ 55	28	15.5	68	25.8		12	14.3	1	5.3	6	10.7	49	46.7	
Smoking status	No	136	75.6	178	68.7	**0.042**	63	75.9	9	50.0	33	61.1	73	70.2	0.161*
	Yes	28	15.6	65	25.1		18	21.7	7	38.9	17	31.5	23	22.1	
	Ex-smoker	16	8.9	16	6.2		2	2.4	2	11.1	4	7.4	8	7.7	
Ethnicity	Caucasian	95	52.8	131	51.4	0.773	41	49.4	7	38.5	25	50.0	58	55.8	0.553
	No caucasian	85	47.2	124	48.6		42	50.6	11	61.5	25	50.0	46	44.2	
Education level[a]	Incomplete elementary school	51	28.3	113	44.1	**0.013**	21	25.3	6	33.3	20	40.0	66	62.9	**<0.001***
	Complete elementary school	24	13.3	33	12.9		12	14.5	1	5.6	6	12.0	14	13.3	
	Incomplete high school	24	13.3	22	8.6		10	12.0	1	5.6	7	14.0	4	3.8	
	Complete high school	59	32.8	72	28.1		34	41.0	9	50.0	14	28.0	15	14.3	
	Incomplete undergraduate degree	7	3.9	5	2.0		2	2.4	0	0.0	3	6.0	0	0.0	
	Complete undergraduate degree	15	8.3	11	4.3		4	4.8	1	5.6	0	0.0	6	5.7	
Marital status	Married	131	72.4	152	57.8	**0.006**	47	56.0	8	42.1	34	60.7	63	60.6	**0.014***
	Single	17	9.4	50	19.0		21	25.0	4	21.1	15	26.8	10	9.6	
	Divorced	24	13.3	38	14.4		11	13.1	4	21.1	6	10.7	17	16.3	
	Widowed	9	5.0	23	8.7		5	6.0	3	15.8	1	1.8	14	13.5	
Monthly income[b]	≤ 1 minimum wage	77	42.8	98	53.3	**0.023***	34	41.0	6	33.3	33	67.3	25	71.4	**<0.001***
	1 - 3 min. wages	96	53.3	75	40.5		41	49.4	10	55.6	16	32.7	8	22.9	
	3 - 5 min. wages	3	1.7	10	5.4		8	9.6	2	11.1	0	0.0	0	0.0	
	5 - 7 min. wages	3	1.7	1	0.5		0	0.0	0	0.0	0	0.0	1	2.9	
	≥ 7 min. wages	1	0.6	1	0.5		0	0.0	0	0.0	0	0.0	1	2.9	

[a]Based on Brazilian educational system. [b]Based on Brazilian minimum wage (approximately U$ 287.00). Data presented as absolute number and percentage. Analysis carried out using the two-tailed Chi-square (X^2) test or *Fisher test, with p<0.05 being adopted as the significance level (SPSS Inc., Chicago, Illinois, USA). HPV (Human Papillomavirus); LSIL (Low-grade squamous intraepithelial lesions); HSIL (High-grade squamous intraepithelial lesions); NL (No lesion); CC (Cervical cancer). Bold values represent statistical significance (*p*<0.05). **Variable containing incomplete data for some participants.
