# Supplementary material for: CTLA4 Haplotype Structures and −318 C>T (rs5742909) Genetic Variant Contribute to the Susceptibility of HPV Infection and Cervical Cancer
Source: Viruses. 2025 Mar 21;17(4):453. doi: 10.3390/v17040453 (PMC12031065; doi:10.3390/v17040453)
Supplement: Supplementary file 1 [file viruses-17-00453-s001.zip › Supplementary Table S2.pdf]

Supplementary Table S2. Association of participant reproductive and sexual behavior characteristics with HPV infection and cervical lesion status.

Variables		HPV				*p*-value	Lesion grade (HPV infected patients)								*p*-value
		Uninfected (n=181)		Infected (n=264)			NL (n=84)		LSIL (n=19)		HSIL (n=56)		CC (n=105)		
		N	%	N	%		N	%	N	%	N	%	N	%	
Oral contraceptive usage**	No	125	69.1	148	56.9	**0.010**	57	68.7	10	52.6	29	52.7	52	50.5	0.073
	Yes	56	30.9	112	43.1		26	31.3	9	47.4	26	47.3	51	49.5	
Condom usage**	No	162	90.0	205	81.0	**0.010**	69	83.1	15	83.3	42	85.7	79	76.7	0.521
	Yes	18	10.0	48	19.0		14	16.9	3	16.7	7	14.3	24	23.3	
Age at menarche (years)**	≤11	39	21.5	53	26.9	0.273	16	21.4	6	31.6	19	33.9	10	26.3	0.875
	12	43	23.8	55	27.9		26	31.0	5	26.3	15	26.8	9	23.7	
	13	47	26.0	38	19.3		15	17.9	4	21.1	11	19.6	8	21.1	
	≥14	52	28.7	51	25.9		25	29.7	4	21.1	11	19.7	11	28.9	
Age at first sexual intercourse (years)**	≤17	87	48.1	162	63.3	**0.002**	48	57.1	15	78.9	39	69.6	60	61.9	0.167
	≥18	94	51.9	94	36.7		36	42.9	4	21.1	17	30.4	37	38.1	
Sexual partners during the lifetime**	1	70	38.9	45	24.2	**0.007**	23	27.7	3	16.7	7	14.6	12	32.4	0.360*
	2	35	19.4	31	16.7		11	13.3	4	22.2	9	18.8	7	18.9	
	3	26	14.4	28	15.1		10	12.0	6	33.3	9	18.8	3	8.1	
	4	13	7.2	19	10.2		9	10.8	2	11.1	5	10.5	3	8.1	
	≥5	36	20.0	63	33.9		30	36.1	3	16.7	18	37.5	12	32.4	
Knowledge about HPV**	No	32	17.8	53	28.0	0.062	19	22.9	4	22.2	12	24	18	47.4	0.107
	Heard about	99	55.0	89	47.1		45	54.2	8	44.4	23	46	13	34.2	
	Yes	49	27.2	47	24.9		19	22.9	6	33.3	15	30	4	18.4	
Knowledge about HPV transmission**	No	18	43.3	97	51.3	0.124	32	38.6	11	91.1	24	48.0	30	78.9	**<0.001**
	Yes	102	56.7	92	48.7		51	61.4	7	38.9	26	52.0	8	21.1	
Previous cervical screening**	No	4	2.2	7	3.7	0.398	4	4.8	0	0.0	0	0.0	3	8.1	0.207*
	Yes	176	97.8	181	96.3		79	95.2	18	100.0	50	100.0	34	91.9	
Familial cervical cancer**	No	158	87.8	168	88.0	0.958	72	86.7	17	94.4	45	86.5	34	89.5	0.906*
	Yes	22	12.2	23	12.0		11	13.3	1	5.6	7	13.5	4	10.5	

Data presented as absolute number and percentage. Analysis carried out using the two-tailed Chi-square (X^2) test or *Fisher test, with $p<0.05$ being adopted as the significance level (SPSS Inc., Chicago, Illinois, USA). HPV (Human Papillomavirus); LSIL (Low-grade squamous intraepithelial lesions); HSIL (High-grade squamous intraepithelial lesions); NL (No lesion); CC (Cervical cancer). Bold values represent statistical significance ($p<0.05$). **Variable containing incomplete data for some participants.
