# Supplementary material for: CTLA4 Haplotype Structures and −318 C>T (rs5742909) Genetic Variant Contribute to the Susceptibility of HPV Infection and Cervical Cancer
Source: Viruses. 2025 Mar 21;17(4):453. doi: 10.3390/v17040453 (PMC12031065; doi:10.3390/v17040453)
Supplement: Supplementary file 1 [file viruses-17-00453-s001.zip › Supplementary Table S3.pdf]

Supplementary Table S3. Association of participants sociodemographic characteristics with HPV infection through adjusted logistic regression.

Variables		HPV + OR (CI95%)	Adjusted *p*-value
Age range (years)	≤ 24	4.673 (1.543-14.153)	**0.006**
	25 – 34	2.049 (0.915-4.588)	0.081
	35 – 44	1.107 (0.523-2.344)	0.791
	45 – 54	0.651 (0.316-1.341)	0.245
	≥ 55	Reference	
Smoking status	No	Reference	
	Yes	0.958 (0.426-0.2.153)	0.917
	Ex-smoker	1.718 (0.686-4.303)	0.248
Education level[a]	Incomplete elementary school	Reference	
	Complete elementary school	0.660 (0.316-1.380)	0.270
	Incomplete high school	0.362 (0.158-0.830)	**0.016**
	Complete high school	0.595 (0.319-1.108)	0.102
	Incomplete undergraduate degree	0.262 (0.064-1.076)	0.063
	Complete undergraduate degree	0.364 (0.117-1.134)	0.081
Marital status	Married	Reference	
	Single	1.791 (0.870-3.688)	0.114
	Divorced	1.620 (0.845-3.107)	0.147
	Widowed	2.336 (0.892-6.120)	0.084
Monthly income[b]	≤ 1 minimum wage	1.430 (0.070-29.259)	0.817
	1 - 3 min. wages	0.944 (0.047-19.052)	0.970
	3 - 5 min. wages	5.325 (0.209-135.590)	0.311
	5 - 7 min. wages	0.453 (0.006-32.737)	0.717
	≥ 7 min. wages	Reference	

[a]Based on Brazilian educational system. [b]Based on Brazilian minimum wage (approximately U$ 287.00). Data were analyzed by logistic regression with $p<0.05$ considered significant (bold) and with "uninfected" group as reference (SPSS Inc., Chicago, Illinois, USA). HPV (Human Papillomavirus); OR (Odds Ratio); CI (confidence interval).
