# Supplementary material for: CTLA4 Haplotype Structures and −318 C>T (rs5742909) Genetic Variant Contribute to the Susceptibility of HPV Infection and Cervical Cancer
Source: Viruses. 2025 Mar 21;17(4):453. doi: 10.3390/v17040453 (PMC12031065; doi:10.3390/v17040453)
Supplement: Supplementary file 1 [file viruses-17-00453-s001.zip › Supplementary Table S4.pdf]

Supplementary Table S4. Association of participant reproductive and sexual behavior characteristics with HPV infection through adjusted logistic regression.

Variables		HPV + OR (CI95%)	Adjusted *p*-value
**Oral contraceptive usage**	No	0.918 (0.580-1.452)	0.714
	Yes	Reference	
**Condom usage**	No	0.776 (0.400-1.508)	0.455
	Yes	Reference	
**Age at first sexual intercourse (years)**	≤17	1.411 (0.901-2.210)	0.133
	≥18	Reference	
**Sexual partners during the lifetime**	1	0.430 (0.239-0.772)	**0.005**
	2	0.563 (0.296-1.072)	0.080
	3	0.655 (0.332-1.294)	0.223
	4	0.900 (0.395-2.050)	0.802
	≥5	Reference	

Data were analyzed by logistic regression with $p<0.05$ considered significant (bold) and with “uninfected” group as reference (SPSS Inc., Chicago, Illinois, USA). HPV (Human Papillomavirus); OR (Odds Ratio); CI (confidence interval).
