# Supplementary material for: CTLA4 Haplotype Structures and −318 C>T (rs5742909) Genetic Variant Contribute to the Susceptibility of HPV Infection and Cervical Cancer
Source: Viruses. 2025 Mar 21;17(4):453. doi: 10.3390/v17040453 (PMC12031065; doi:10.3390/v17040453)
Supplement: Supplementary file 1 [file viruses-17-00453-s001.zip › Supplementary Table S5.pdf]

Supplementary Table S5. Association of participant sociodemographic, reproductive and sexual behavior characteristics with cervical lesion groups through adjusted logistic regression.

Variables		Lesion grade (HPV infected patients)					
		LSIL	Adj. *p*-value	HSIL	Adj. *p*-value	CC	Adj. *p*-value
		OR (CI95%)		OR (CI95%)		OR (CI95%)	
Age range (years)	≤ 24	22.375 (1.554-322.123)	**0.022**	1.106 (0.246-4.968)	0.896	-	0.974
	25 – 34	4.414 (0.335-58-195)	0.259	2.051 (0.488-8.614)	0.327	1.539 (0.229-10.3200	0.657
	35 – 44	2.793 (0.214-36.433)	0.433	2.151 (0.545-8.891)	0.274	1.471 (0.329-6.564)	0.613
	45 – 54	4.536 (0.355-57.909)	0.245	1.512 (0.376-6.091)	0.561	0.579 (0.125-2.677)	0.485
	≥ 55	Reference		Reference		Reference	
Education level[a]	Incomplete elementary school	Reference		Reference		Reference	
	Complete elementary school	0.240 (0.014-3.997)	0.320	-	0.998	0.389 (0.050-3.058)	0.370
	Incomplete high school	0.030 (0.001-1.144)	0.059	-	0.998	0.128 (0.013-1.316)	0.084
	Complete high school	0.066 (0.002-1.850)	0.110	-	0.998	-	0.996
	Incomplete undergraduate degree	0.411 (0.349-0.028)	0.411	-	0.998	0.050 (0.006-0.457)	**0.008**
	Complete undergraduate degree	-	-	-	-	-	0.998
Marital status	Married	Reference		Reference		Reference	
	Single	0.092 (0.013-0.656)	**0.017**	3.630 (0.382-34.516)	0.262	0.437 (0.097-1.964)	0.280
	Divorced	0.130 (0.014-1.185)	0.070	2.627 (0.244-28.331)	0.426	-	0.994
	Widowed	0.427 (0.054-3.356)	0.418	2.859 (0.257-31.742)	0.392	0.521 (0.092-2.945)	0.460
Knowledge about HPV	No	0.987 (0.192-5.083)	0.987	0.578 (0.180-1.862)	0.359	1.126 (0.224-5.645)	0.886
	Heard about	0.593 (0.144-2.444)	0.470	0.531 (0.196-1.437)	0.212	0.327 (0.071-1.509)	0.152
	Yes	Reference		Reference		Reference	

[a]Based on Brazilian educational system. Data were analyzed by logistic regression with $p<0.05$ considered significant (bold) and with "no lesion" group as reference (SPSS Inc., Chicago, Illinois, USA). HPV (Human Papillomavirus); LSIL (Low-grade squamous intraepithelial lesions); HSIL (High-grade squamous intraepithelial lesions); CC (Cervical cancer); OR (Odds Ratio); CI (confidence interval); Adj. (Adjusted). Some categories of the variables did not complete the logistic regression, probably due to the sample size.
